# Ataque De Nervios: A Case Report

**DOI:** 10.1192/bjo.2022.371

**Published:** 2022-06-20

**Authors:** Sitki Ustun

**Affiliations:** North East London NHS Foundation Trust, London, United Kingdom

## Abstract

**Aims:**

Ataque de nervios is a culture-bound syndrome among individuals of Latin descent.The case study presents an ataque de nervios case triggered by unique stressors in a transgender female of Mexican origin. This case also highlights the critical features of ataque de nervios, and discusses the challenges in diagnosis, classification and management.

**Methods:**

47-year-old transgender (male to female) woman was admitted to an acute female inpatient unit following a presentation of erratic behaviour and a collapse episode under mental health act. During her observation and assessment period, multiple ataque episodes were observed with characteristic features triggered by gender phobic comments. After ruling out the organic causes and ruling out other differentials, she was diagnosed with ataque de nervios. She completed her treatment and continues to remain on remission.

**Results:**

The case presents a rather recognized presentation of ataque de nervios, however, the unique triggers rooted from her gender identity are more relevant to the current 21st century social context which is also an important ongoing discussion topic in psychiatry. The themes are also good presentation of the evolving psychosocial context of the specialty.

**Conclusion:**

Further study is needed to examine the relationship between ataque de nervios and gender identity, as well as the relationship to cultural, demographic, environmental, and personality factors.

